# Semaphorin 3A promotes the long-term persistence of human SVF-derived microvascular networks in engineered grafts

**DOI:** 10.3389/fbioe.2024.1396450

**Published:** 2024-08-21

**Authors:** Juan M. Schwager, Nunzia Di Maggio, Andrea Grosso, Abeelan Rasadurai, Nadja Minder, Jeffrey A. Hubbell, Elisabeth A. Kappos, Dirk J. Schaefer, Priscilla S. Briquez, Andrea Banfi, Maximilian G. Burger

**Affiliations:** ^1^ Regenerative Angiogenesis Laboratory, Department of Biomedicine, Basel University Hospital and University of Basel, Basel, Switzerland; ^2^ Department of Plastic, Reconstructive, Aesthetic and Hand Surgery, Basel University Hospital, Basel, Switzerland; ^3^ Pritzker School of Molecular Engineering, University of Chicago, Chicago, IL, United States; ^4^ Department of Clinical Research, Medical Faculty, University of Basel, Basel, Switzerland; ^5^ Department of General and Visceral Surgery, Medical Center–University of Freiburg, Freiburg, Germany

**Keywords:** stromal-vascular fraction, adipose tissue, angiogenesis, semaphorin 3A, vessel stabilization, fibrin

## Abstract

**Introduction:**

The stromal vascular fraction (SVF) of human adipose tissue is an attractive cell source for engineering grafts with intrinsic vascularization potential, as it is rich in vasculogenic progenitors. However, in order to maintain their functional perfusion it is important to promote the *in vivo* stabilization of newly assembled microvascular networks. We previously found that Semaphorin 3A (Sema3A) promotes the rapid stabilization of new blood vessels induced by VEGF overexpression in skeletal muscle. Here we investigated whether Sema3A could promote the assembly, connection to circulation and persistence of human SVF-derived microvascular networks in engineered grafts.

**Methods:**

Recombinant Sema3A was engineered with a transglutaminase substrate sequence (TG-Sema3A) to allow cross-linking into fibrin hydrogels. Grafts were prepared with freshly isolated human SVF cells in fibrin hydrogels decorated with 0, 0.1 or 100 μg/ml TG-Sema3A and implanted subcutaneously in immune-deficient mice.

**Results:**

After 1 week in vivo, the assembly of human-derived networks was similar in all conditions. The outer part of the grafts was populated by blood vessels of both human and mouse origin, which formed abundant hybrid structures within a common basal lamina. About 90% of human-derived blood vessels were functionally connected to the host circulation in all conditions. However, in the control samples human vessels were unstable. In fact, they significantly regressed by 6 weeks and could no longer be found by 12 weeks. In contrast, a low Sema3A dose (0.1 μg/ml) promoted further human vascular expansion by about 2-fold at 6 weeks and protected them from regression until 12 weeks. From a mechanistic point of view, the stabilization of SVF-derived vessels by 0.1 μg/ml of Sema3A correlated with the recruitment of a specific population of monocytes expressing its receptor Neuropilin-1.

**Discussion:**

In conclusion, Sema3A is a potent stimulator of *in vivo* long-term persistence of microvascular networks derived from human SVF. Therefore, decoration of matrices with Sema3a can be envisioned to promote the functional support of tissue engineered grafts.

## 1 Introduction

An important and unresolved challenge in tissue engineering and regenerative medicine is the lack of rapid vascularization of clinical-sized tissue substitutes after *in vivo* implantation. In fact, invasion by the host vasculature is a slow process limited to tens of microns per day (Utzinger et al., 2015), leading to necrosis in the deeper layers of the grafts and impairing adequate restoration of tissue function (Scheufler et al., 2008; Gianni-Barrera et al., 2020). To accelerate graft vascularization, different methods have been explored (Goncalves et al., 2021). Among them, an attractive strategy comprises endowing the engineered constructs with an intrinsic vasculogenic capacity, whereby microvascular networks may self-assemble inside the graft within a few days after implantation and ensure effective functional perfusion from the host circulation at a minimum by 7 days. This is in contrast with the process of angiogenesis, whereby vessels grow out of pre-existing networks and gradually invade the graft. Endothelial cells can undergo vasculogenesis, i.e., self-assemble into microvascular networks, when cultured in a suitable 3D matrix, such as fibrin, but this requires the interaction with pericyte-like support cells like mesenchymal cells of different origin (Verseijden et al., 2010; Rohringer et al., 2014). The stromal vascular fraction (SVF) of human adipose tissue is a particularly attractive cell source for this purpose, as it is rich in both vasculogenic and mesenchymal progenitors (Miranville et al., 2004; Scherberich et al., 2010).

However, newly formed vessels are unstable and tend to regress in the absence of sustained vascular endothelial growth factor (VEGF) signaling for about 4 weeks (Dor et al., 2002; Ozawa et al., 2004; Tafuro et al., 2009). It is therefore important to promote the *in vivo* stabilization of microvascular networks formed by vasculogenic self-assembly in engineered grafts in order to maintain their functional perfusion. We previously identified the neural-guidance molecule Semaphorin 3A (Sema3A) as a key mediator of vascular stabilization after VEGF delivery to skeletal muscle, through the recruitment of a subpopulation of Neuropilin1 (NRP-1)-expressing monocytes (NEM) and the activation of TGF-β1 signaling (Groppa et al., 2015). Therefore, here we investigated the ability of recombinant Sema3A to promote the stabilization and long-term persistence of human SVF-derived self-assembled micro-vascular networks *in vivo*. To this end, SVF cells were embedded in fibrin matrices decorated with an engineered version of Sema3A, which was fused to an octapeptide substrate of the coagulation transglutaminase Factor XIIIa (TG-Sema3A) in order to allow its covalent cross-linking into the fibrin hydrogel upon fibrinogen polymerization (Sacchi et al., 2014). Thus, the cross-linked factor cannot passively diffuse, but it is immobilized in the matrix both *in vitro* and upon *in vivo* implantation (Ehrbar et al., 2004; Sacchi et al., 2014). The release profile *in vivo* depends on the rate of enzymatic degradation of the fibrin matrix by invading cells (Sacchi et al., 2014) and, until that point, the factor is protected from degradation and remains biologically active (Largo et al., 2014).

## 2 Materials and methods

### 2.1 SVF isolation

Stromal vascular fraction (SVF) cells were isolated as previously described (Guven et al., 2011). Lipoaspirates or excision fat samples were obtained during routine liposuction, after informed consent and following protocol approval by the ethical committee of the local Government (Permit number 78/07 of the Ethikkommission beider Basel, Kanton Basel-Stadt, Basel, Switzerland). SVF cells were isolated from 6 healthy donors ranging in age from 28 to 56 years. Adipose tissue samples were digested in 0.075% collagenase type 2 (Worthington) on an orbital shaker for 45 min at 37°C. The digested tissues were centrifuged at 300 *g* for 10 min. The pellet, containing the stromal vascular fraction (SVF), was rinsed once with PBS (Gibco), resuspended in alpha-minimal essential medium (α-MEM, Gibco) and filtered through a 100 μm and 70 μm strainer (BD Falcon). SVF nucleated cells were counted with 0.01% Crystal Violet (Sigma) in PBS.

### 2.2 Recombinant TG-Sema3A production and purification

The amino acid sequence of mouse Sema3A (NCBI Reference Sequence: NP_033178.2) is 96% identical with that of human Sema3A (NCBI Reference Sequence: NP_006071.1). Further, signalling activity has been shown to be widely conserved across different species (mouse, human, rat and chicken). To produce the engineered cross-linkable form of mouse Semaphorin 3A (Sema3A), before insertion into the expression vector pXLG (provided from the Protein Expression Core Facility (PECF) of the Ecole Polytechnique Federale de Lausanne (EPFL), Switerland), the cDNA for mouse Sema3A was amplified by PCR using primers designed to add the transglutaminase (TG) substrate sequence NQEQVSPL, including the 8 N-terminal residues of α2-plasmin inhibitor (α2PI_1–8_, hereafter referred to as TG-), onto the N-terminus of the amplified cDNA (Schense et al., 2000). An 8x histidine was then also added at the N-terminus. The engineered protein was expressed in mammalian cells, Human Embryonic Kidney (HEK)-293 F (ThermoFischer Scientific, Waltham MA, United States). Cells were transfected with the TG-Sema3A containing plasmid using PEI-Max and cultured for 7 days under shaking conditions at 37°C, 5% CO_2_ in FreeStyle 293-F medium. The supernatant was then collected and purified using an HisTrap column and Äkta Pure FPLC machine (GE Heatlhcare, Chicago IL, United States) using standard procedures according to the manufacturer. Sema3A monomers and dimers were then separated using size exclusion with a HiLoad 16/60 Superdex 75-pg column (GE healthcare, Chicago IL, United States). Fractions corresponding to Sema3A dimers were pooled, concentrated with Amicon tubes and filtered through a 0.22 μm filter. Sema3A dimers purity was assessed by SDS/PAGE and found to be >99%. Endotoxin level was under 0.05 EU/mg of protein after measurement with the HEK-Blue mTLR4 assay (Invivogen, San Diego, California, United States).

### 2.3 Recombinant TG-Aprotinin production and purification

The engineered cross-linkable form of aprotinin was produced as previously described (Burger et al., 2022). Briefly, the cDNA for bovine aprotinin was modified adding an 6x histidine tag and a thrombin cleavage site at its N-terminus, and the cDNA of the TG substrate sequence NQEQVSPL at its C-terminus. The cDNA was subcloned into a pXLG vector for the expression in HEK-293F mammalian cells. HEK-293F cells were cultured in suspension for 7 days after transient transfection with polyethylenimine (PEI). The supernatant was then collected and purified via immobilized metal affinity chromatography (HisTrap columns, GE healthcare, Chicago, Illinois, United States) using an Äkta Pure FPLC system (GE healthcare, Chicago, Illinois, United States). The purified proteins were then dialyzed and the histidine tag was cleaved using thrombin (50 U/mg protein) for 24 h at room temperature. The proteins were purified again using HisTrap and benzamidine columns (GE healthcare, Chicago, Illinois, United States) to remove the cleaved his-tag and thrombin. The purified TG-aprotinin was dialyzed against TBS, concentrated using Amicon tubes, filtered through a 0.22 μm filter and stored at −80°C.

### 2.4 Generation and *in vivo* implantation of fibrin constructs

Engineered constructs were generated using 60 mm^3^ of silicate-substituted calcium phosphate granules (1–2 mm in size, Actifuse^®^; Apatech-Baxter, Elstree, United Kingdom), 2 × 10^6^ of SVF cells and 60 µL fibrin gel. The fibrin hydrogel was prepared by mixing 25 mg/mL human fibrinogen (plasminogen-, von Willebrand Factor-, and fibronectin-depleted; Milan Analytica AG, Rheinfelden, Switzerland), 3 U/mL factor XIII (CSL Behring, King of Prussia, Pennsylvania, United States), and 6 U/mL thrombin (Sigma-Aldrich, St. Louis, Missouri, United States) with 2.5 mM Ca^2+^ in 4-(2-hydroxyethyl)-1-piperazineethanesulfonic acid (Hepes, Lonza, Basel, Switzerland). The engineered constructs displayed a spherical shape with a diameter of about 6 mm. Decoration with 51 μg/mL of TG-Aprotinin and 0.1 or 100 μg/mL of TG-SEMA3A were obtained by adding the engineered proteins to the cross-linking enzymes solution before mixing with fibrinogen. Fibrin was allowed to polymerize at 37°C for 10 min after mixing with all the other components before *in vivo* implantation. The engineered constructs were implanted subcutaneously (4 constructs/animal) in 5-week old female nude mice (CD1-*Foxn1* ^*nu*^, Charles-River, Sulzfeld, Germany). Animals were treated in agreement with Swiss legislation and according to a protocol approved by the Veterinary Office of Canton Basel-Stadt (permission #1797). After 1, 6 and 12 weeks, mice were sacrificed by inhalation of CO_2_ and constructs were explanted.

### 2.5 Histological processing and immunofluorescence tissue staining

After harvesting, constructs were washed with PBS and fixed overnight at 4°C with freshly prepared 1% paraformaldehyde (Sigma-Aldrich, St. Louis, Missouri, United States) in PBS. They were then decalcified in a solution containing 7% w/v EDTA (0.5M, pH 8, Sigma-Aldrich, St. Louis, Missouri, United States) and 10% w/v sucrose (Sigma-Aldrich, St. Louis, Missouri, United States) and incubated at 37°C on an orbital shaker. The decalcification solution was changed daily until the samples were fully decalcified, as estimated by the degree of sample stiffness (for a maximum of 20 days). Finally, the samples were embedded in OCT compound (CellPath LTD., Newtown, United Kingdom), frozen in freezing 2-methylbutane (isopentane) (Sigma-Aldrich, St. Louis, Missouri, United States) and sectioned with a cryostat with a thickness of 10 µm.

The following primary antibodies were used for immunofluorescence staining: rat anti-mouse CD31 (clone MEC 13.3, BD Bioscience, San Jose, California, United States) at 1:100; mouse anti-human CD31 (clone JC70, Santa Cruz, Dallas, United States) at 1:200; rat anti-mouse CD11b (clone M1/70, Thermofisher, Waltham, Massachusset, United States) at 1:200; rabbit polyclonal anti-Neuropilin 1 (Abcam, Cambridge, United Kingdom) at 1:200, rabbit polyclonal anti-Laminin (Abcam, Cambridge, United Kingdom, 11575) at 1:200. Secondary antibodies labeled with fluorescent fluorochromes (Invitrogen, Thermo Fisher Scientific, Waltham, Massachusetts, United States) were used at 1:200.

Images were acquired with a Nikon Ti2 Eclipse microscope (Nikon, Tokyo, Japan) and a Nikon Ti2 inverted microscope (Nikon, Egg, Switzerland) equipped with a X-Light V3 Spinning disk confocal unit (CrestOptics) (50 μm pinhole size with 250 μm spacing), a Celesta Light Engine (LumenCor), and a Prime 95B (Photometrics) camera. All image measurements were performed with FIJI software (ImageJ, http://fiji.sc/Fiji), NIS-Elements (Nikon, Tokyo, Japan) and QuPath software (v 0.4.4 https://qupath.github.io/). All subsequent quantifications were performed by 3 independent observers who were blinded to the sample identities.

### 2.6 Angiogenesis

Immunostaining for CD31 was used to asses vascular invasion and vascular density. Reconstructions of whole sections from the central part of each sample were acquired and the invaded area was measured by tracing the area occupied by mouse blood vessels (mCD31^+^ structures) and expressed as percentage of the total graft area. To quantify vessel density at week 1, at least 15 images were acquired per sample within the invaded areas and vessel length density (VLD) was measured tracing manually the total length of vessels, distinguishing those of mouse and human origin, in the fields and by normalizing it to the field area (mm/mm^2^). The same images were also used to quantify the amount of perfused vessels, which were identified by the presence of red blood cells (RBC) within the lumen. VLD at 6 and 12 weeks was quantified on 15 randomly acquired images, covering all the area of the tissue section, since constructs were completely invaded by these time-points. Every quantified image covered a field of 0.3 mm^2^ and therefore a total area of 4.5 mm^2^ was quantified for each sample. Since the total area of the histological section through the center of each graft had an area of approximately 20–25 mm^2^, about 20% of the total area was quantified in each sample, in order to minimize the effects of non-homogeneous vessel distribution within the constructs.

### 2.7 Quantification of NRP1^+^ monocytes (NEM)

The number of NEM recruited to the constructs after 1 week *in vivo* was quantified by immunostaining for NRP-1 in combination with a monocyte marker (CD11b) and the DNA dye DAPI. Images of whole sections for each condition were acquired and a concentric layer of 1 mm depth from the external surface was traced. NRP^+^/CD11^+^ cells were automatically detected and counted by using the open-source software QuPath v0.4.4. The DAPI channel was used for cell identification and pixel classifiers were generated for the NRP-1 and CD11b channels. A composite classifier was created for the colocalization of the three individual channels.

### 2.8 Statistics

Data are presented as mean ± standard error of the mean (SEM). The significance of differences was assessed with the GraphPad Prism software (Version 9.5.1, GraphPad Software, San Diego, California, United States). The normal distribution of all data sets was tested and, depending on the results, multiple comparisons were performed with the parametric one-way analysis of variance (ANOVA) followed by the Dunnet test, or with the non-parametric Kruskal–Wallis test followed by Dunn’s post-test. Differences were considered statistically significant if *p* < 0.05.

## 3 Results

### 3.1 Sema3A does not affect the initial self-assembly of hSVF cells into microvascular networks

**FIGURE 1:**
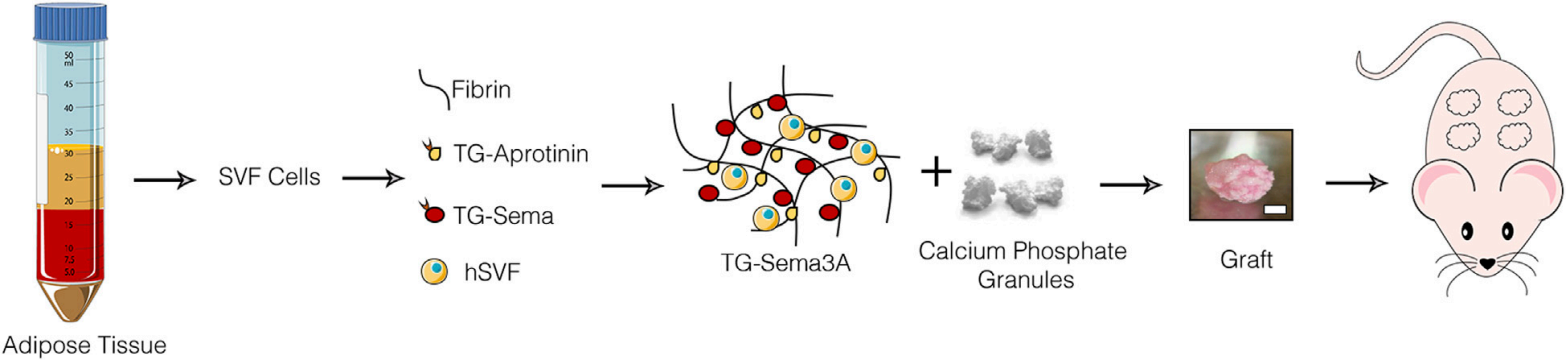
Study design. Grafts were generated by combining human adipose tissue-derived SVF cells with calcium phosphate granules in fibrin hydrogels decorated with TG-Sema3A and implanted ectopically in immunodeficient mice; scale bar = 2 mm.

Grafts were generated with fibrin decorated with two doses of TG-Sema3a (0.1 and 100 μg/mL) or without the recombinant factor as control, together with 2 × 10^6^ freshly isolated human adipose tissue-derived stromal-vascular fraction cells (SVF) (Figure 1). The two doses were chosen to be far apart, in order to cover a wide biologically relevant spectrum, as previously found for the factor VEGF (Grosso et al., 2023). In order to provide the grafts with structural stability and persistence similar to a typical application like for bone tissue engineering, silicate-substituted calcium phosphate granules (Actifuse^®^) were also included (Burger et al., 2022). Further, to ensure a slow degradation of the fibrin matrix over a period of at least 4 weeks, a TG-version of the fibrinolysis inhibitor aprotinin was included at the concentration of 51 μg/mL, as previously optimized (Sacchi et al., 2014). Constructs were then implanted in subcutaneous pockets in immune-deficient nude mice and harvested after 1, 6 or 12 weeks.

**FIGURE 2:**
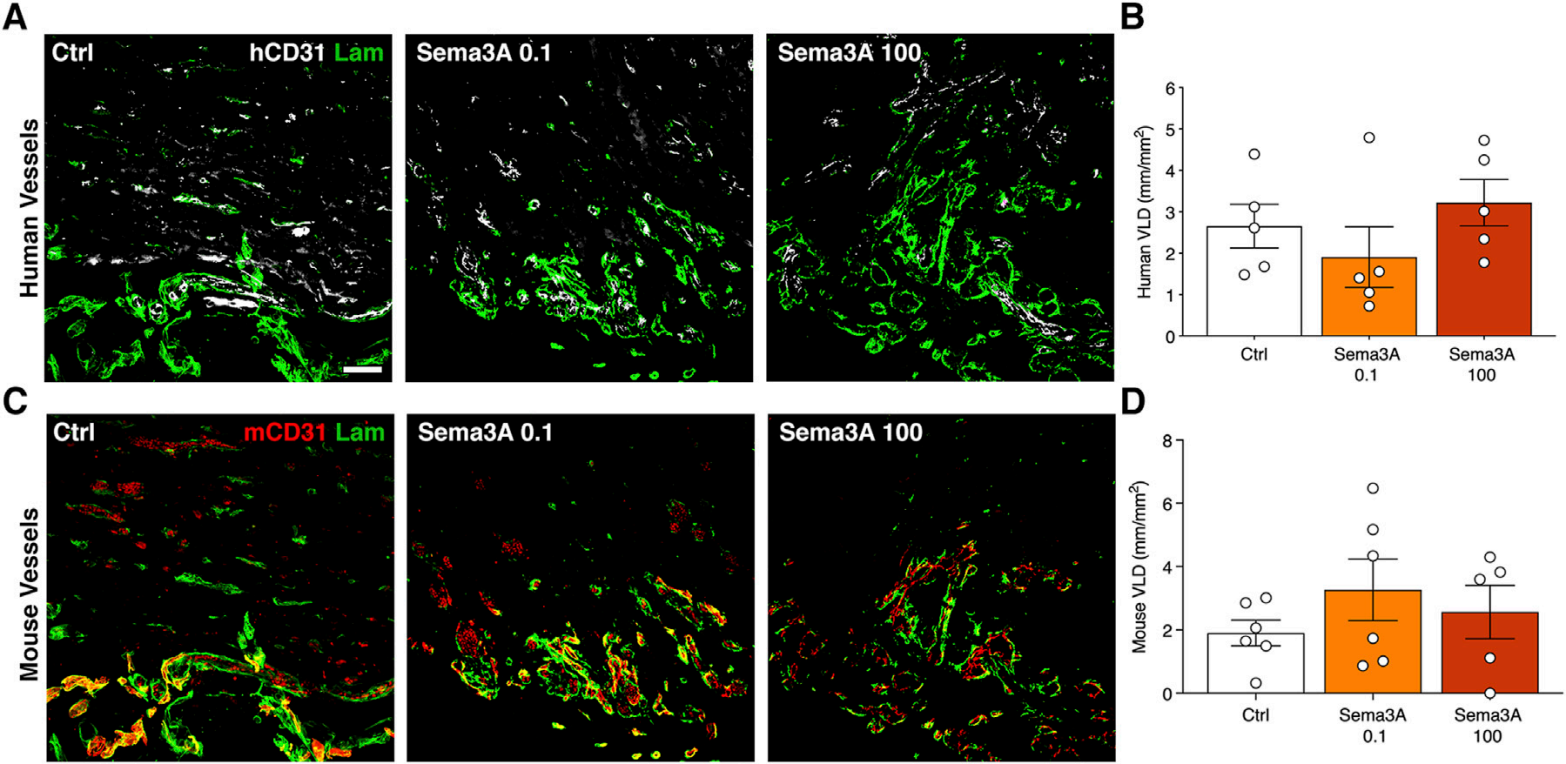
Angiogenesis after 1 week of *in vivo* implantation. Immunostaining of human-derived **(A)** and mouse-derived **(C)** vessels (hCD31 in white, mCD31 in red and laminin in green). Quantification of human **(B)** and mouse **(D)** vessels, expressed as vessel length density (VLD) = millimeters of vessel length per square millimeter of tissue area (mm/mm^2^). Values are expressed as mean ± s.e.m. Scale bar = 50 µm.

After 1 week the human SVF cells had efficiently self-assembled into microvascular networks throughout the constructs, as evidenced by specific immunostaining for human endothelium (hCD31) embedded within a laminin-positive basal lamina (Figure 2A). VLD quantification showed that the efficiency of this initial self-assembly step was not affected by the presence of Sema3A at any dose (Figure 2B; Control = 2.7 ± 0.5 mm/mm^2^; Sema3A 0.1 = 1.9 ± 0.7 mm/mm^2^; Sema3A 100 = 3.2 ± 0.6 mm/mm^2^, *p* = n.s.). On the other hand, host-derived vessels could be identified by staining for mouse-specific CD31 and were found to have started invading the outer part of the grafts (Figure 2C). The density of host vascular networks was also not affected by Sema3A (Figure 2D; Control = 1.9 ± 0.4 mm/mm^2^; Sema3A 0.1 = 3.3 ± 1.0 mm/mm^2^; Sema3A 100 = 2.6 ± 0.9 mm/mm^2^, *p* = n.s.).

### 3.2 hSVF-derived microvascular networks connect efficiently with invading host vessels and are functionally perfused by 1 week

**FIGURE 3:**
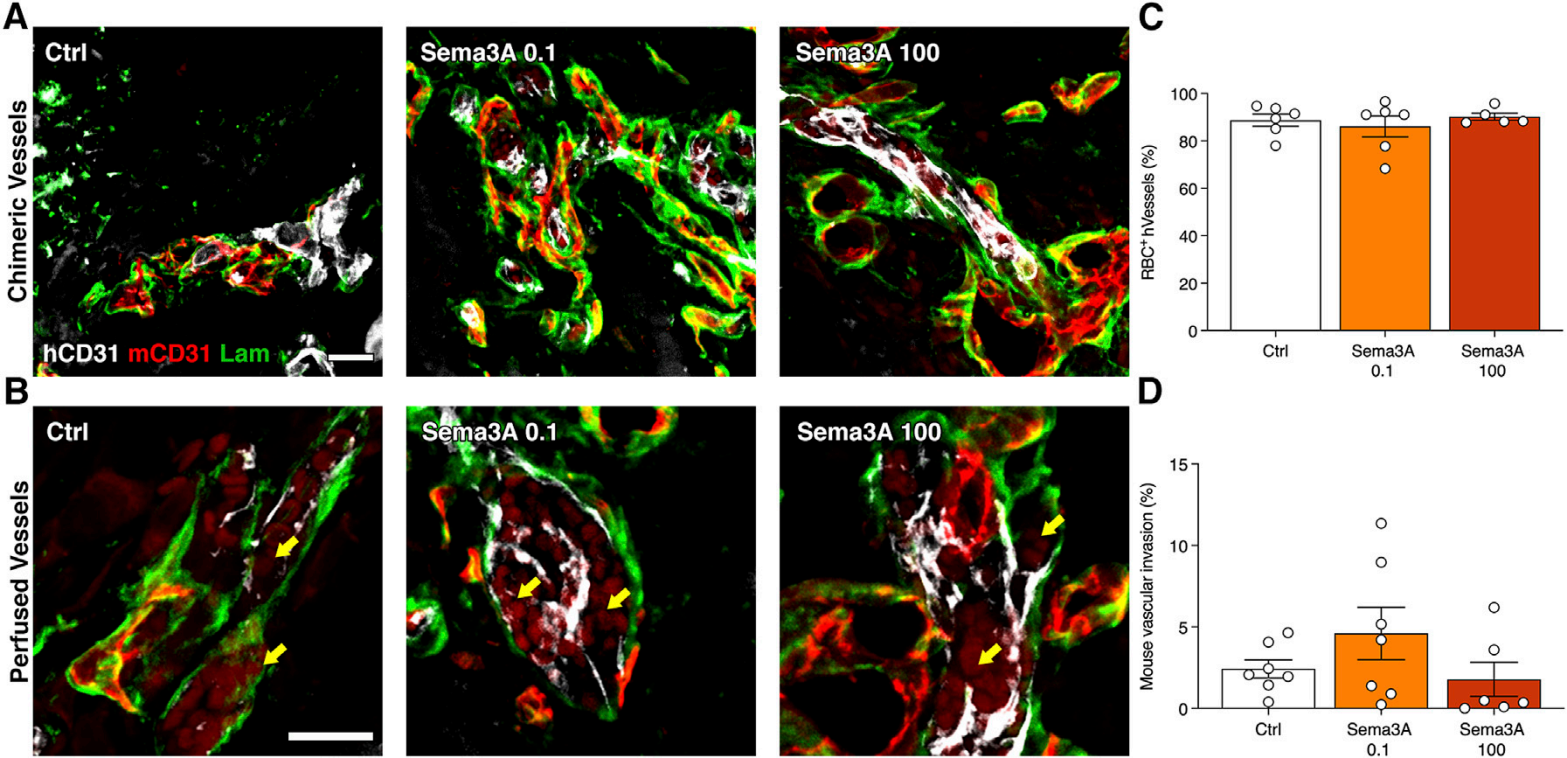
Human blood vessel perfusion and mouse blood vessel invasion after 1 week. **(A)** Representative images of hybrid blood vessels containing both human (hCD31 in white) and mouse (mCD31 in red) endothelium within a common basal lamina (laminin in green). **(B)** Representative images of hybrid vessels connected to the circulation as evidenced by the presence of red blood cells (RBC) (yellow arrows). **(C)** Quantification of the perfused human blood vessels (percentage of the total). **(D)** Quantification of the areas invaded by mouse blood vessels (percentage of the total section area). Values are expressed as mean ± s.e.m. Scale bars **(A)** = 50 μm; **(B)** = 20 µm.

After 1 week, the outer part of the grafts was populated by blood vessels of both human and mouse origin. By co-staining with species-specific anti-CD31 antibodies, it was found that in this outer portion human and mouse microvascular networks were connected in all conditions, as shown by abundant hybrid vascular structures containing both human and mouse endothelium within a common basal lamina (Figure 3A). Furthermore, these chimeric vessels were functionally connected to the host circulation, as evidenced by the presence of red blood cells within the lumens (Figure 3B). Quantification of the amount of human-derived blood vessels that were perfused by red blood cells showed that the self-assembled networks were about 90% functional in all conditions (Figure 3C; Control = 88.8 ± 2.6%; Sema3A 0.1 = 86.2 ± 4.4%; Sema3A 100 = 90.2 ± 1.5%, *p* = n.s.). Conversely, we investigated whether the ingrowth of host blood vessels could be influenced by Sema3A. Histological quantification of the percentage of graft areas invaded by mouse vascular networks showed similar results in all conditions (Figure 3D; Control = 2.4 ± 0.6%; Sema3A 0.1 = 4.6 ± 1.6%; Sema3A 100 = 1.8 ± 1.0%, *p* = n.s.). Together with the data reported in Figure 2D, these results show that Sema3A did not affect the initial host vascular growth inside the grafts, neither in its invasion speed nor in its density.

### 3.3 0.1 μg/mL Sema3A specifically expand human microvascular networks by 6 weeks

**FIGURE 4:**
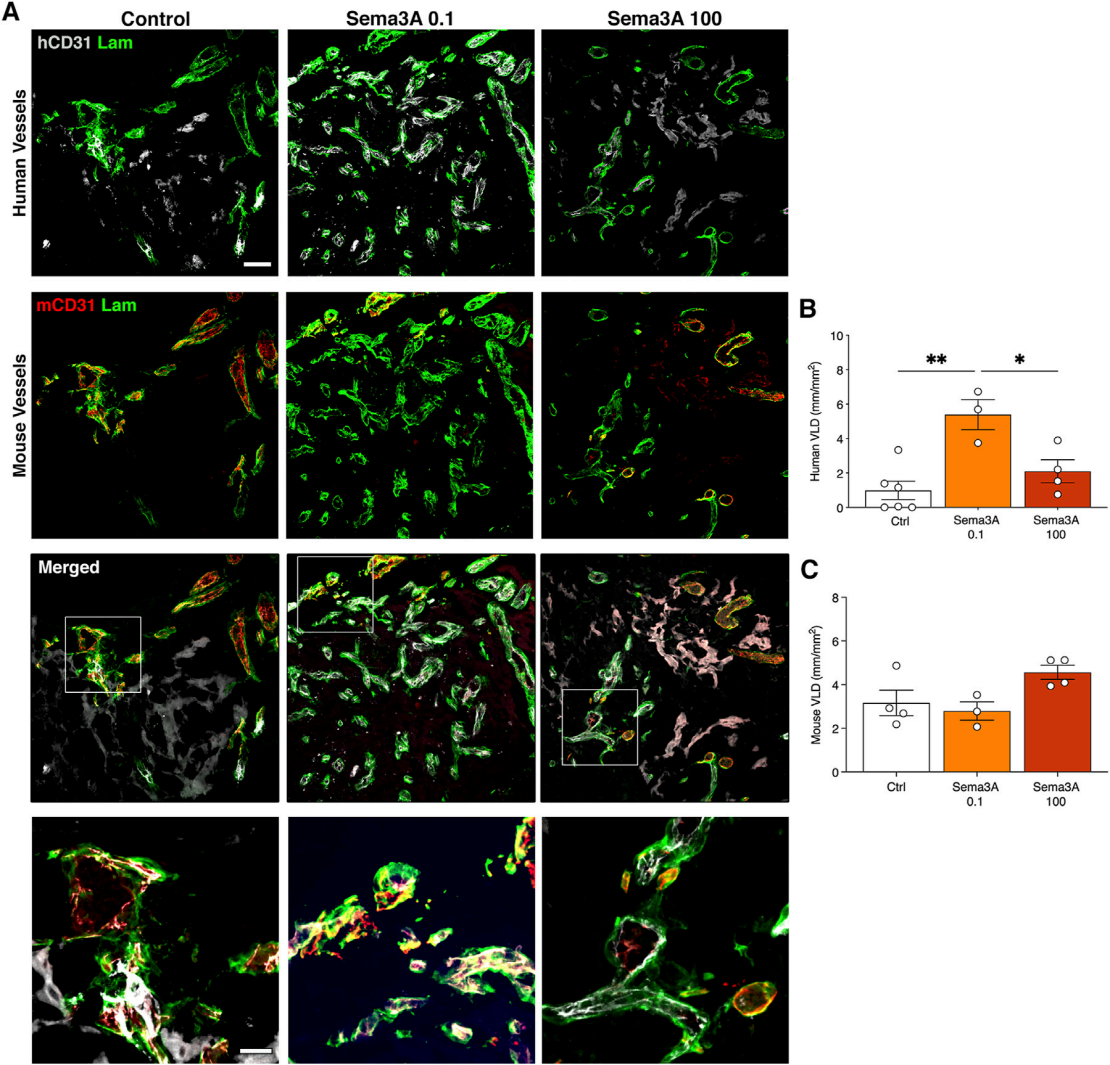
Graft vascularization after 6 weeks *in vivo*. **(A)** Immunostaining of human (hCD31 in white) and mouse (mCD31 in red) vessels covered by basal lamina (laminin in green). The lower panels show higher magnification images of hybrid vessels. Quantification of human **(B)** and mouse **(C)** vessels, expressed as vessel length density (VLD) = millimeters of vessel length per square millimeter of tissue area (mm/mm^2^). Values are expressed as mean ± s.e.m.; * = *p* < 0.05, ** = *p* < 0.01. Scale bars = 50 µm (upper panels) and 20 µm (lower panels).

After 6 weeks *in vivo*, graft invasion by the host vessels was completed and the microvascular networks were comprised of both human and mouse components, which were still connected and giving rise to clear hybrid vessels similar to those at 1 week (Figure 4A). Vessel density quantification showed that in the control condition the human vascularization was diminished compared to the 1-week time-point (VLD 1 week = 2.7 ± 0.5 mm/mm^2^ vs. 6 weeks = 1.0 ± 0.5 mm/mm^2^, *p* = 0.05). In contrast, the presence of TG-Sema3A caused a significant expansion of the human SVF-derived vasculature at a low dose of 0.1 μg/mL (1 week = 1.9 ± 0.7 mm/mm^2^ vs. 6 weeks = 5.3 ± 0.9 mm/mm^2^, *p* < 0.05), but not at the higher dose (TG-Sema3A 100 μg/mL = 2.1 ± 0.7 mm/mm^2^, *p* < 0.05 vs. TG-Sema3A 0.1 μg/mL and *p* = n.s vs. Control). This effect appeared to be specific for the self-assembled human vascular networks. In fact, the invading mouse vascularization was not affected by the presence of TG-Sema3A (Figure 4C; Control = 3.2 ± 0.6 mm/mm^2^; Sema3A 0.1 = 2.8 ± 0.4 mm/mm^2^; Sema3A 100 = 4.6 ± 0.3 mm/mm^2^, *p* = n.s.).

### 3.4 0.1 μg/mL Sema3A protects human microvascular networks from regression until 12 weeks

**FIGURE 5:**
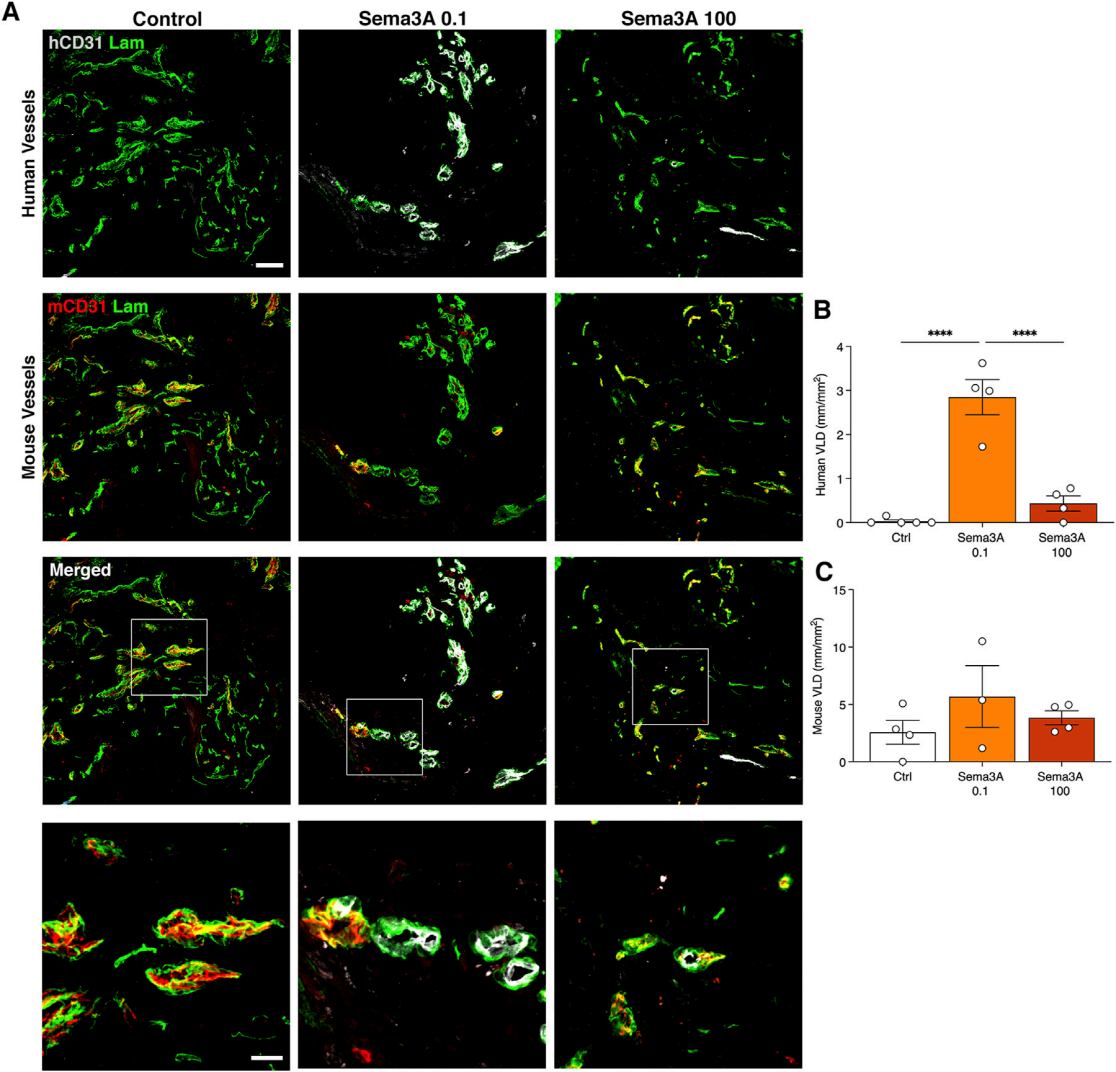
Graft vascularization after 12 weeks *in vivo*. **(A)** Immunostaining of human (hCD31 in white) and mouse (mCD31 in red) vessels covered by basal lamina (laminin in green). The lower panels show higher magnification images of hybrid vessels. Quantification of human **(B)** and mouse **(C)** vessels, expressed as vessel length density (VLD) = millimeters of vessel length per square millimeter of tissue area (mm/mm^2^). Values are expressed as mean ± s.e.m.; *** = *p* < 0.001, **** = *p* < 0.0001. Scale bars = 50 µm (upper panels) and 20 µm (lower panels).

By 12 weeks *in vivo* the self-assembled human vascular networks had completely regressed in the control condition but were still clearly visible in the presence of TG-Sema3A, especially at the low dose of 0.1 μg/mL, whereas mouse-derived vessels were present in all conditions (Figure 5A). Correspondingly, hybrid human-mouse vascular structures were not detected in the absence of TG-Sema3A. Quantification of vessel densities confirmed the specific protective effect of 0.1 μg/mL of TG-Sema3A on the human vascular component (Figure 5B; Control = 0.03 ± 0.03 mm/mm^2^; Sema3A 0.1 = 2.9 ± 0.4 mm/mm^2^; Sema3A 100 = 0.4 ± 0.2 mm/mm^2^, *p* < 0.0001), but not on the mouse vessels (Figure 5C; Control = 2.6 ± 1.0 mm/mm^2^; Sema3A 0.1 = 5.9 ± 2.7 mm/mm^2^; Sema3A 100 = 3.8 ± 0.6 mm/mm^2^, *p* = n.s.).

### 3.5 0.1 μg/mL Sema3A specifically promotes the early recruitment of Neuropilin-1+ monocytes

**FIGURE 6:**
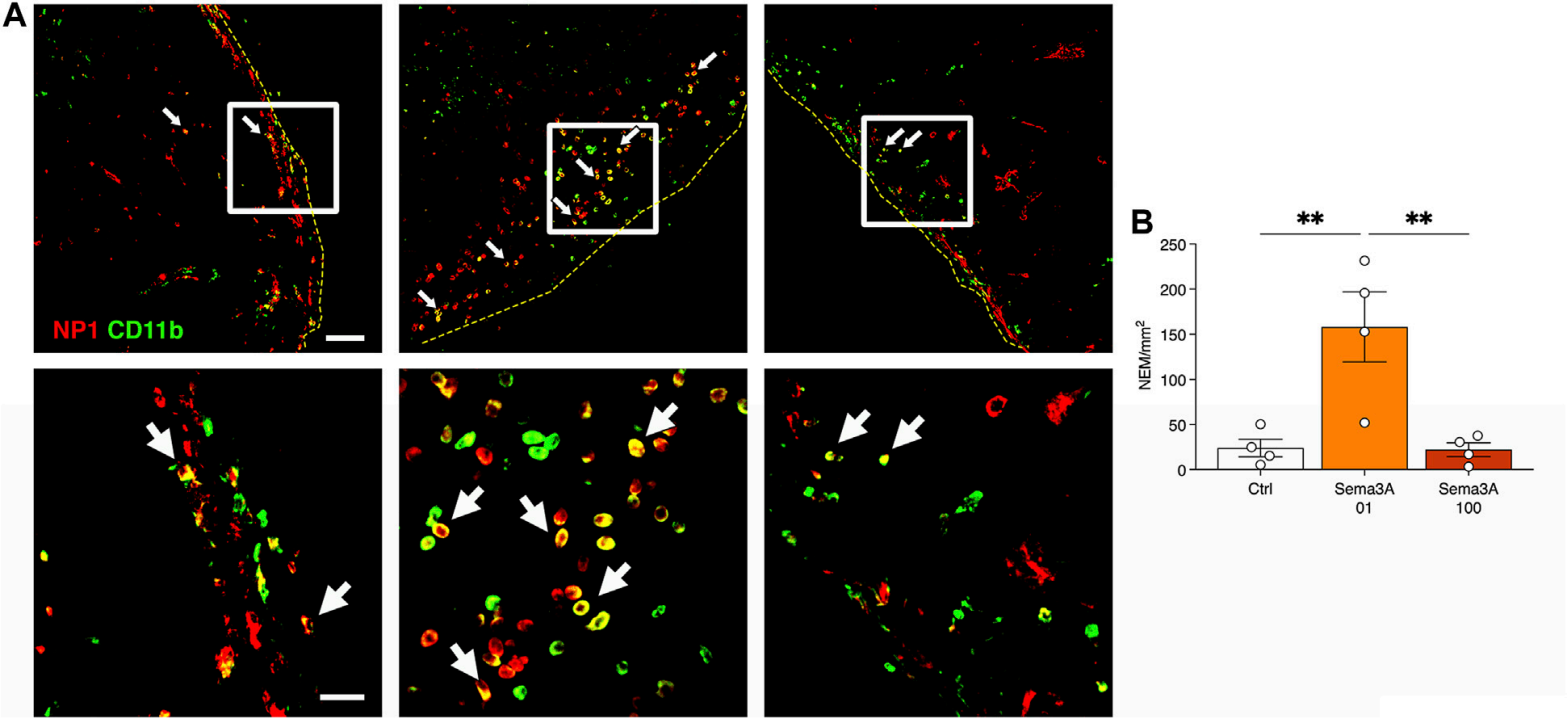
Early recruitment of NRP-1^+^ monocytes (NEM) after 1 week *in vivo*. **(A)** Representative images of graft sections stained for NRP1 (in red) and CD11b (in green). NEM appear yellow as they are positive for both markers (white arrows). Yellow dotted lines indicate the border of the graft. Lower panels show higher magnifications of the areas indicated by white squares in the upper panels. **(B)** Quantification of the number of NEM per square millimeter of tissue area. Values are expressed as mean ± s.e.m.; ** = *p* < 0.001. Scale bars = 50 µm (upper panels) and 20 µm (lower panels).

We previously found that endothelial-derived Sema3A promotes the stabilization and survival of VEGF-induced angiogenesis in skeletal muscle through the early recruitment of a specific population of NRP-1-expressing monocytes (NEM) (Groppa et al., 2015). Therefore, we investigated NEM recruitment in the grafts 1 week after *in vivo* implantation by co-staining for NRP-1 and the monocyte marker CD11b (Figure 6A). Endothelial cells were positive for NRP-1, but not for CD11b, while classic monocytes were CD11b^+^ and NRP1^-^. Double-positive NEM could be identified in the outer layer of the grafts, in the areas that were invaded by mouse vessels and therefore accessible to the circulating cells. A quantification of their frequency revealed that NEM recruitment was significantly greater in the grafts with 0.1 μg/mL of TG-Sema3A compared to both controls and the higher Sema3A dose (Figure 6B; Control = 24.0 ± 9.7 NEM/mm^2^; Sema3A 0.1 = 158.1 ± 38.9 NEM/mm^2^; Sema3A 100 = 22.1 ± 7.7 NEM/mm^2^, *p* < 0.01). These data show that the frequency of NEM recruited to the grafts correlated closely with the improvement of long-term persistence of the self-assembled human microvascular networks by a low dose of 0.1 μg/ml TG-Sema3A.

## 4 Discussion

Here we found that Sema3A specifically promotes the *in vivo* long-term persistence of self-assembled human SVF-derived microvascular networks in engineered grafts, without affecting either their initial assembly or the stabilization of host mouse-derived vascularization.

It is interesting to contrast Sema3A effects on self-assembled networks, which were protected from regression, and on the invading host vasculature, which instead did not require Sema3A supplementation in order to persist. This observation is unlikely to depend on the species identity of the Sema3A protein. In fact, the amino acid sequence of mouse Sema3A (NCBI Reference Sequence: NP_033178.2) is 96% identical with that of human Sema3A (NCBI Reference Sequence: NP_006071.1) and signalling activity has been shown to be widely conserved across different species (mouse, human, rat and chicken). This is reinforced by the observation that the murine Sema3A that was immobilized in the fibrin matrices functionally affected both mouse and human cells, as it stimulated the recruitment of murine Nrp1+ monocytes (NEM, Figure 6) and the stabilization of human blood vessels (Figures 4, 5).

Pericytes are the main cells responsible for promoting the stabilization and persistence of newly formed vessels, through both cell contact-dependent and paracrine signalling (Siekmann, 2023). However, pericyte association does not differ among these two vascularization modalities. In fact, it has been shown that human SVF cells in fibrin hydrogels self-assemble into mature pericyte-covered microvascular networks both *in vitro* and *in vivo* (Klar et al., 2014). On the other hand, mouse microvascular structures invading similar fibrin hydrogels have been also shown to be mature and associated with pericytes (Sacchi et al., 2014). This discrepancy therefore suggests that there may be functional differences in the ability of SVF-derived murals cells and microvascular pericytes to protect newly formed networks from regression. Uncovering the molecular nature of such functional differences will require unbiased analysis of the transcriptomic profile of these mural cell populations at single cell resolution.

However, more recently it has become clear that the process of vascular stabilization does not depend only on pericytes. In fact, a complementary role has been described for a sub-population of monocytes that specifically express the Sema3A receptor Neuropilin-1, named NEM. We previously showed that stabilization of VEGF-induced capillary networks in skeletal muscle requires the recruitment of these monocytes by Sema3A produced by the endothelium and their secretion of TGF-β1, with activation of SMAD2/3 signalling (Groppa et al., 2015). Indeed, here we found that the stabilization of SVF-derived vessels by a moderate dose of Sema3A specifically correlated with a significant recruitment of NEM in the grafts (Figure 6), suggesting that they may play a role in the observed effects of Sema3A. It is unclear why the effects on both NEM recruitment and human vessel stabilization are lost with a greater dose of Sema3A (100 μg/mL) and this observation needs further investigation, as it suggests the existence of a more complex signalling network, potentially involving secondary players or negative feedback loops. However, non-linear dose-effect functions have been previously described for other factors. For example, using a similar factor-decorated fibrin platform, we have recently found that VEGF promotes the acquisition of a pro-osteogenic function by endothelium at a specific dose, but this is lost at higher doses through dose-dependent inhibition of Notch signalling (Grosso et al., 2023). Therefore, these data suggest that NEM recruitment by Sema3A plays a role in promoting the *in vivo* persistence of SVF-derived self-assembled vascular networks. Further, this observation supports a general role for NEM in the stabilization of newly formed vessels generated by different mechanisms. In fact, while the SVF-derived vessels are generated by the process of vasculogenic self-assembly, the microvascular networks in skeletal muscle were formed by the mechanism of intussusceptive (or splitting) angiogenesis (Groppa et al., 2015).

The lack of influence by Sema3A on the initial step of vascular assembly by SVF cells is also in agreement with the previous observations in the context of intussusceptive angiogenesis in skeletal muscle (Groppa et al., 2015). Those results, in fact, also showed that Sema3A and NEM recruitment did not affect the initial expansion of microvascular networks, but only their long-term persistence independently of sustained VEGF signalling, further confirming that the specific roles of Sema3A and NEM are shared across forms of vascular growth.

The work presented here focused on the effects of Sema3A on self-assembled vascular networks in isolation, in order avoid confounding factors while addressing the specific engineering of a potentially universal “vasculogenic module”. In fact, the generation of a self-assembled and stable microvascular network, which can persist long-term *in vivo*, can be envisioned as a stand-alone component, i.e., a “module”, that can be subsequently associated with different tissue-specific progenitors in order to promote specific regeneration. Sema3A is a pleiotropic factor, with known functions in disparate biological processes besides angiogenesis, such as neurogenesis, osteogenesis, cancer progression and immunology (Roth et al., 2009). On the other hand, the use of SVF cells has been investigated both pre-clinically and clinically for a variety of applications, the most investigated of which can be grouped in two major categories: skeletal tissue regeneration and anti-inflammatory treatments to favour spontaneous repair (Andia et al., 2019). It is promising that the known functions of Sema3A in these contexts appear potentially synergistic with the desired clinical outcomes.

1) In skeletal regeneration, Sema3A has an osteoprotective function. In fact, it has been shown that it directly promotes bone formation during development, by both stimulating osteoblast differentiation and impairing osteoclast formation (Hayashi et al., 2012; Li et al., 2017). Both functions depend on Sema3A binding to the Neuropilin-1/Plexin A1 complex receptor. Genetic modelling of this signalling axis showed that both *Sema3a* deletion and removal of the Sema3A-binding domain from the Nrp1 receptor caused loss of bone tissue, with increased osteoclastic activity and reduced bone deposition. Mechanistically, the Sema3A/Nrp1 signalling regulates osteoblasts and osteoclasts differently, as it activates the canonical Wnt/β-catenin pathway in the first case and instead suppresses osteoclast differentiation through the Rho A signalling pathway;
2) Sema3A has also been found to have several functions in immune regulation and autoimmune diseases (Kiseleva and Rutto, 2022). Of particular interest for the combination with SVF in anti-inflammatory applications is its ability to direct macrophage polarization from the classically activated and pro-inflammatory M1 phenotype to the alternatively activated and pro-regenerative M2 phenotype (Teng et al., 2017). Further, Nrp1 activation by another Semaphorin 3 protein (Sema3B) has been found to promote pro-resolving polarization in macrophages from rheumatoid arthritis patients (Martinez-Ramos et al., 2023). Therefore, the prevailing activity of the Sema3/Nrp1 axis in macrophages appears directed towards stimulating the resolution of inflammation and promoting tissue repair.

Fibrin is a widely used hydrogel material for tissue regeneration. In fact, the precursor fibrinogen is an abundant protein in plasma and therefore can be easily isolated from each patient for autologous use (Breen et al., 2009). Furthermore, fibrin is the universal regeneration matrix during physiological tissue repair, as damage always causes the deposition of a transient fibrin-rich matrix, which provides an ideal substrate for cell adhesion and migration, as well as growth factor presentation (Martino et al., 2015). Upon mixing with other mechanically suitable materials, fibrin can also be bioprinted for the engineering of both soft and hard tissues (de Melo et al., 2020). Lastly, fibrin is an already FDA-approved biomaterial for clinical use as a surgical sealant and hemostatic agent. Since fibrin concentration and the stiffness of the resulting hydrogels can adversely influence the efficacy of microvascular network formation (Rao et al., 2012; Muhleder et al., 2018), such parameters should be carefully adapted and balanced with the specific needs of envisioned applications.

In conclusion, these data suggest that the combination of SVF cells and matrices decorated with a specific low dose of Sema3A holds promise for the long-term persistence of self-assembled microvascular networks in engineered grafts. This can be envisioned as a “vasculogenic module” that could be combined with a variety of specialized parenchymal cells/progenitors for the regeneration of different tissues.

## Data Availability

The raw data supporting the conclusions of this article will be made available by the authors, without undue reservation.
